# Unique Cell Type-Specific Junctional Complexes in Vascular Endothelium of Human and Rat Liver Sinusoids

**DOI:** 10.1371/journal.pone.0034206

**Published:** 2012-04-03

**Authors:** Cyrill Géraud, Konstantin Evdokimov, Beate K. Straub, Wiebke K. Peitsch, Alexandra Demory, Yvette Dörflinger, Kai Schledzewski, Astrid Schmieder, Peter Schemmer, Hellmut G. Augustin, Peter Schirmacher, Sergij Goerdt

**Affiliations:** 1 Department of Dermatology, Venereology and Allergology, University Medical Center and Medical Faculty Mannheim, University of Heidelberg, and Center of Excellence in Dermatology, Mannheim, Germany; 2 Department of Pathology, University Clinic Heidelberg, University of Heidelberg, Germany; 3 Department of General and Transplantation Surgery, Ruprecht-Karls-University, Heidelberg, Germany; 4 Joint Research Division Vascular Biology, German Cancer Research Center, Heidelberg, Germany; 5 Medical Faculty Mannheim, University of Heidelberg, Mannheim, Germany; Katholieke Universiteit Leuven, Belgium

## Abstract

Liver sinusoidal endothelium is strategically positioned to control access of fluids, macromolecules and cells to the liver parenchyma and to serve clearance functions upstream of the hepatocytes. While clearance of macromolecular debris from the peripheral blood is performed by liver sinusoidal endothelial cells (LSECs) using a delicate endocytic receptor system featuring stabilin-1 and -2, the mannose receptor and CD32b, vascular permeability and cell trafficking are controlled by transcellular pores, i.e. the fenestrae, and by intercellular junctional complexes. In contrast to blood vascular and lymphatic endothelial cells in other organs, the junctional complexes of LSECs have not yet been consistently characterized in molecular terms. In a comprehensive analysis, we here show that LSECs express the typical proteins found in endothelial adherens junctions (AJ), i.e. VE-cadherin as well as α-, β-, p120-catenin and plakoglobin. Tight junction (TJ) transmembrane proteins typical of endothelial cells, i.e. claudin-5 and occludin, were not expressed by rat LSECs while heterogenous immunreactivity for claudin-5 was detected in human LSECs. In contrast, junctional molecules preferentially associating with TJ such as JAM-A, B and C and zonula occludens proteins ZO-1 and ZO-2 were readily detected in LSECs. Remarkably, among the JAMs JAM-C was considerably over-expressed in LSECs as compared to lung microvascular endothelial cells. In conclusion, we show here that LSECs form a special kind of mixed-type intercellular junctions characterized by co-occurrence of endothelial AJ proteins, and of ZO-1 and -2, and JAMs. The distinct molecular architecture of the intercellular junctional complexes of LSECs corroborates previous ultrastructural findings and provides the molecular basis for further analyses of the endothelial barrier function of liver sinusoids under pathologic conditions ranging from hepatic inflammation to formation of liver metastasis.

## Introduction

Liver sinusoidal endothelial cells (LSECs) form a fenestrated monolayer at the inner side of the liver sinusoids constituting a barrier between blood flow and hepatocytes facing the perisinusoidal space of Disse [1], [2], [3]. Leukocyte recruitment upon liver injury [4], [5], [6] as well as liver colonization by metastatic tumor cells [7], [8] are actively influenced by LSECs. The unique morphology as well as the microenvironment-dependent molecular differentiation of LSECs [19] define the organ-specific features of this transendothelial barrier. Despite recent advances in understanding extravasation of inflammatory and tumor cells in liver sinusoids [6], the intercellular junctions between LSECs that considerably contribute to regulating hepatic transmigration have not yet been sufficiently characterized in molecular terms.

The most remarkable morphological hallmark of LSECs is the presence of fenestrae that are arranged in clusters being referred to as sieve plates. The fenestrae of LSECs form open pores that lack a diaphragm; they contribute substantially to the high permeability of LSECs compared to other microvascular endothelial cells [9], [10], [11]. Besides diffusion through the fenestrae, LSECs actively support uptake and degradation as well as transendothelial transfer of macromolecules by their high endocytic capacity. Endocytic clearance of soluble macromolecules from the circulation is mediated by specialized endocytic receptors [12] including the stabilins identified by us previously [13], [14]. The hepatic clearance function of LSECs is highly important for the homeostasis of the whole organism protecting distant organs such as the kidney from noxious blood factors [14].

Another important morphological feature of LSECs is their lack of an ultrastructurally identifiable basement membrane. The major molecular constituents of the vascular basal lamina in general such as collagen IV, collagen VI, fibronectin, and tenascin are detectable as amorphous material in the perisinusoidal space of Disse [15]. LSECs correspondingly express a distinct repertoire of integrins to interact with this extracellular matrix in the space of Disse [15], [16]. In line with this, we and others have shown that the phenotype and functional activity of LSECs are strongly influenced by the extracellular matrix and by the surrounding hepatic cell populations such as Kupffer cells and hepatic stellate cells with which LSECs intermingle in the wall of the liver sinusoids [17], [18], [19].

**Table 1:** Human patients involved in the study.

Patient Nr	Sex	Age	Diagnosis	Pathologic diagnosis of analyzed liver specimens
1	female	59	Colorectal carcinoma with liver metastasis	No significant liver pathology
2	male	66	Colorectal carcinoma with liver metastasis	No significant liver pathology
3	male	72	Acinar cell carcinoma of the salivary gland with liver metastasis	No significant liver pathology
4	male	46	Hepatocellular carcinoma (pT2, pN0 (0/2), Mx, G2);	Complete micronodular liver cirrhosis
5	male	55	Cholangiocarcinoma	Moderate liver fibrosis
6	male	64	Hepatocellular carcinoma (pT1, pN0 (0/1), Mx, G2)	Portal and pericellular liver fibrosis with septa formation

**Table 2:** Primary antibodies used in the study.

Antibody	Catalog Nr	Supplier
anti-VE-cadherin polyclonal rabbit IgG	ALX-210-232	Enzo Life Sciences
anti-VE-cadherin monoclonal mouse IgG_2a_ (clone BV9)	ab7047	Abcam
anti-VE-cadherin polyclonal goat IgG	AF1002	R&D Systems
anti-VE-cadherin polyclonal goat IgG	sc-6458	Santa Cruz Biotechnology
anti-VE-cadherin polyclonal rabbit IgG	V1514	Sigma
anti-E-cadherin monoclonal mouse IgG_2a_,κ (clone 36/E-Cadherin)	610182	BD Transduction Laboratories
anti-N-cadherin monoclonal mouse IgG_1_ (clone 32)	610921	BD Transduction Laboratories
anti-α-catenin polyclonal rabbit IgG	C2081	Sigma
anti-β-catenin monoclonal mouse IgG_1_ (clone 14)	610153	BD Transduction Laboratories
anti-p120 catenin monoclonal mouse IgG_1_ (clone 98/pp120)	610133	BD Transduction Laboratories
anti-plakoglobin monoclonal mouse IgG		custom made
anti-JAM-A polyclonal rabbit Ig	36-1700	Invitrogen
anti-Claudin-5 monoclonal mouse IgG_1_ (clone 4C3C2)	35-2500	Invitrogen
anti-Occludin monoclonal mouse IgG_1_,κ (clone OC-3F10)	33-1500	Invitrogen
anti-ZO-1 polyclonal rabbit Ig	40-2200	Invitrogen
anti-ZO-2 polyclonal rabbit Ig	71-1400	Invitrogen
anti-LYVE-1 polyclonal rabbit Ig	103-PA50	RELIATech
anti-Stabilin-2 monoclonal mouse IgG		custom made
anti-human CD32 polyclonal goat IgG	AF1330	R&D Systems

Electron microscopy studies have identified junctional complexes between cytoplasmic processes of adjacent LSECs; these junctional complexes, however, did not precisely correspond to typical adherens junctions (AJ) and even less so to typical tight junctions (TJ) [20], [21], [22]. These findings were confirmed *in vitro* in isolated human LSECs [23]. In line with these ultrastructural ambiguities, it is still a matter of debate whether VE-cadherin (Cdh5), the cadherin defining AJ in vascular endothelium, is indeed expressed in LSECs. VE-cadherin was shown to be expressed in LSECs of human embryos and fetuses during antenatal development and the first postnatal week [16] as well as in murine LSECs analyzed by FACS [24]. Others, however, have reported that VE-cadherin was barely detectable in human liver samples [25] and absent from isolated human LSECs [23].

Regarding TJ molecules, LSECs of nontumorous areas in samples of human hepatocellular carcinoma were shown to contain claudin-5, the typical transmembrane component of TJ in vascular endothelium [26]. By contrast, vascular endothelial junctional adhesion molecule (JAM-B/VE-JAM/JAM-2), a member of the junctional adhesion molecule (JAM) family also typically localizing to TJ could not be clearly detected in human liver at the mRNA level [27], [28]. In addition, mouse liver sinusoids were found to be negative for JAM-C as revealed by immunohistochemistry [29]. However, endothelial cell-selective adhesion molecule (ESAM), a TJ molecule closely related to the JAMs, was shown to be expressed in LSECs and to influence leukocyte transmigration upon ischemia-reperfusion injury in the liver [6]. As a consequence, the controversial findings regarding the ultrastructure and the molecular composition of the junctional complexes in LSECs have led Lalor and colleagues to suggest that classical adherens and tight junctions are absent in LSECs [30].

While the overall structure and molecular architecture of AJ and TJ initially studied in epithelial tissues have been found to be conserved in vascular endothelium, the particular organization of TJ and AJ varies along the vascular tree and between blood vascular and lymphatic endothelium [31]. Recent studies have identified tissue-specific forms of intercellular junctions, among these mixed-type junctions containing molecular components of both AJ and TJ [32]. The endothelial cells of the initial lymphatics and of the lymph node sinuses, for example, exhibit a special combination of junctional molecules that usually occur as physically separated in AJ and TJ [32], [33]. Moreover, there is molecular infidelity regarding the junctional complexes of AJ and TJ during establishment and maintenance of cell-cell contacts; ZO-1, for example, may be found in either AJ or TJ or both 31,34,35. Furthermore, studies in polarized epithelial cell lines demonstrated that JAMs, initially identified in TJ, can participate in the formation of various types of intercellular junctional complexes [28]. As a consequence, distinct endothelial barrier functions such as control of vascular permeability and leukocyte trafficking have been shown to be determined by the organ-specific molecular architecture of intercellular junctions in blood and lymphatic vascular endothelium [32], [33], [36].

Given this variability of intercellular junctions in different endothelial cell types at different developmental stages and along the vascular tree and given the inconsistent and incomplete findings regarding junctions in LSECs, the present study was designed to comprehensively analyze and clearly define the molecular composition of intercellular junctional complexes in LSECs. We show here that LSECs are equipped with complex junctions of distinct molecular composition and discuss how these results pertain to the sinusoidal barrier function in liver development, physiology and disease.

## Materials and Methods

### Animals

Sprague-Dawley rats were purchased from Janvier (Le Genest-St-Isle, France) and received humane care according to the guidelines of the National Institutes of Health (NIH).

### Human Tissues

Clinical and histological data of human liver tissue specimens are shown in Table 1. The tissue was taken at least 5 cm away from the tumor and was snap-frozen in liquid nitrogen. All human tissue specimens were provided by the tissue bank of the National Center for Tumor Diseases (NCT, Heidelberg, Germany) in accordance to the ethics committee of the University of Heidelberg (ethics proposal 207/2005).

### Cell Line

Rat hepatoma McA-RH7777 cell line was purchased from the American Type Culture Collection and maintained in Dulbecco's Modified Eagle Medium (Invitrogen, Darmstadt, Germany) supplemented with 10% Fetal Bovine Serum (Biochrom, Berlin, Germany) and 1% Sodium pyruvate (Sigma, Hamburg, Germany).

**Table 3:** Primers used in the study.

Primer Name	Sequence 5′-3′
Rn_Cdhn5_F2229	ACGGCTACGAGGGCACAGAGTCCAT
Rn_Cdhn5_R2432	TGCAATGAGATTGGGTCCCCAGGCC
Rn_Cdhn5_F1735	TGGAACCGGCACGCTAACAGTG
Rn_Cdhn5_R2090	ATACTGCTGGCCGGGCATCCAT
Rn_Cdhn2_F380	TGGCGGCCTTGCTTCAGGCATCTCT
Rn_Cdhn2_R602	GCGTACACTGTGCCGTCCTCATCCA
Rn_Cdhn1_F771	AGGCTGGCTGAAAGTGACGCAGCCT
Rn_Cdhn1_R980	ACGGAGGTTCCTGGAAGAGCGCCTT
Rn_Occludin_F1361	ACAGGTGGCGAGTCCTGCGA
Rn_Occludin_R1584	GCAGCAGCCATGTACTCTTCGCTC
Rn_Claudin5_F51	GCACCAGAATCAGCCCCCAACCCA
Rn_Claudin5_R280	AGTCGTCTGCGCCGTCACGATA
Rn_JAMA_F2	CCCAGCGCAGTGGATAGCGA
Rn_JAMA_R219	ACAGGGCAACTTGACAGAGTCGT
Rn_JAMB_F689	AATCCAAAGGGCGGCGCACACAGGA
Rn_JAMB_R919	GCCACACGCAGAAATGACGAAGGCC
Rn_JAMC_F79	TGCTGCTCTTCAGGGGCTGCGTGAT
Rn_JAMC_R301	AACACATCTGTGCGACCGGCCAGGT
Rn_bActin_258_FW	GGCACCACACTTTCTACAATGA
Rn_bActin_644_BW	TCTCTTTAATGTCACGCACGAT

### Isolation, Purification, and Culture of LSECs and LMECs

Cells were isolated and purified as described previously [17], [19], [37]. Briefly, for LSECs isolation the rat liver was perfused via the portal vein with Collagenase P (Roche Applied Science, Mannheim, Germany) containing buffer for 8 minutes followed by dissection of the liver. The organ lysates were cleared from tissue debris by centrifugation and then centrifuged at 400 g to yield a cell pellet. The resuspended cell pellet was then separated on a Percoll (GE Healthcare, Munich, Germany) density gradient (25%/50%). Finally the cells found in the interphase of the Percoll gradient were subjected to MACS sorting with a monoclonal antibody against stabilin-2 and the monoclonal SE-1 antibody. Purity after sorting was confirmed by FACS with directly-labeled antibodies against Stabilin-2 and CD11b resulting in >95% Stabilin-2 positive, CD11b negative cells. LSECs were plated on collagen-coated dishes and cultured using a mixture of EBM-2 (Cambrex, Wiesbaden, Germany) and Willams’ E (Invitrogen, Darmstadt, Germany) growth medium, containing EGM Single-Quots (Cambrex, Wiesbaden, Germany), 0.2% bovine serum albumin (BSA), 10 ng/mL hepatocyte growth factor (HGF), and 1% ITS media supplement (Sigma, Hamburg, Germany) at 37°C in a humidified incubator (5% CO_2_). For lung microvascular endothelial cells (LMECs) isolation dissected rat lungs were digested in Collagenase IV (Worthington, Lakewood, NJ) solution and subjected to MACS sorting with a mouse anti rat CD31 antibody. Experiments were approved by the animal ethics committee in Baden-Wuerttemberg (Regierungspräsidium Karlsruhe AZ:35-9185.82A-35/07).

**Figure 1:**
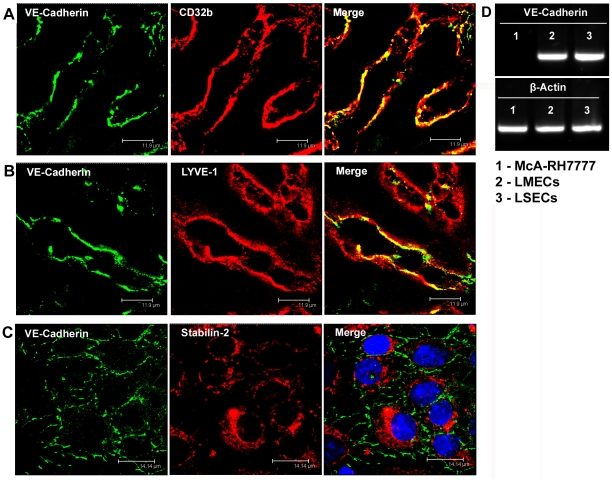
VE-cadherin is expressed in liver sinusoidal endothelial cells in rats and humans. (A) Immunofluorescent co-staining of human liver cryosections with anti-VE-cadherin (green) and anti-CD32b (red) antibodies. (B) Immunofluorescent co-staining of rat liver cryosections with anti-VE-cadherin (green) and anti-LYVE-1 (red) antibodies. (C) Immunofluorescent co-staining of isolated rat LSECs with anti-VE-cadherin (green) and anti-Stabilin-2 (red) antibodies. Toto3 (blue) was used to counterstain the cell nuclei. Images were acquired using laser scanning confocal microscopy. Bars 11.9 µm (A, B), 14.14 µm (C). (D) Reverse transcriptase-PCR with mRNA isolated from rat hepatoma McA-RH7777 cell line (1), freshly isolated rat LMECs (2), and freshly isolated rat LSECs (3). Primers specific for VE-cadherin or β-actin were used.

**Figure 2:**
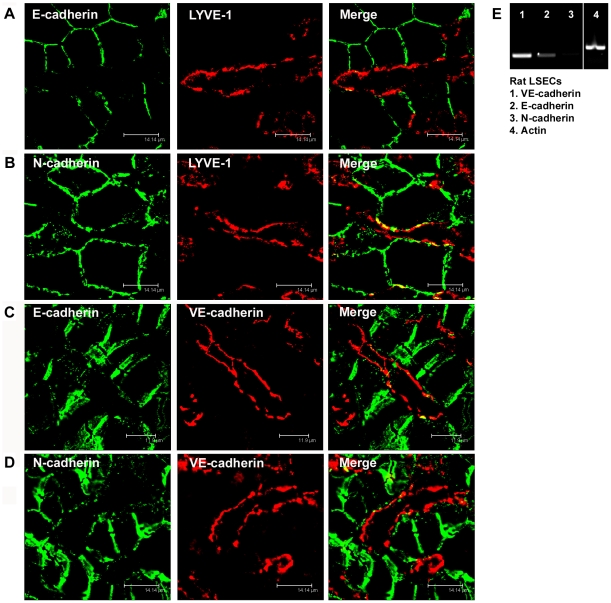
E- and N-cadherin are absent in liver sinusoidal endothelial cells. (A, B) Immunofluorescent co-staining of rat liver cryosections with anti-E-cadherin (A, green) or anti-N-cadherin (B, green) and anti-LYVE-1 (A, B, red) antibodies. (C, D) Immunofluorescent co-staining of human liver cryosections with anti-E-cadherin (C, green) or anti-N-cadherin (D, green) and anti-VE-cadherin (C, D, red) antibodies. Images were acquired using laser scanning confocal microscopy. Bars 14.14 µm (A, B, D), 11.9 µm (C). (E) Reverse transcriptase-PCR with mRNA of freshly isolated rat LSECs. Primers specific for VE-cadherin (1), E-cadherin (2), N-cadherin (3) or β-actin (4) were used.

### Antibodies, Immunofluorescence, and Confocal Microscopy

Acetone-fixed cryosections and 4%-paraformaldehyde-fixed cells on coverslips were blocked with 5% BSA, incubated with first antibodies, followed by appropriate secondary antibodies. First antibodies are listed in Table 2. Secondary antibodies were Cy3-, DyLight 488-, DyLight 649- and Cy5-conjugated donkey anti-rabbit, anti-mouse, or anti-goat IgG (Dianova, Hamburg, Germany) as well as AlexaFluor 488-coupled anti-mouse and anti-rabbit IgG (MoBiTec, Göttingen, Germany). Specimens were analyzed by laser scanning spectral confocal microscopy (Leica, Heidelberg, Germany). Excitation and detection wave lengths were as follows: 488 nm excitation and 518 nm emission maximum for DyLight 488, 543 nm excitation and 570 nm emission maximum for Cy3, and 633 nm excitation and 673 nm emission maximum for DyLight 649 and Cy5. Images were acquired in a sequential mode. Co-localization analysis was performed using ImageJ software as described elsewhere [38]. Five different antibodies against independent epitopes of VE-cadherin (Table 2) were tested in immunohistochemistry of rat and human liver samples. Immunofluorescent stainings of rat and human liver samples presented below were performed with anti-VE-cadherin polyclonal goat IgG (R&D Systems, Wiesbaden, Germany) and anti-VE-cadherin polyclonal rabbit IgG (Enzo Life Sciences, Lörrach, Germany) respectively. The data were confirmed in at least three independent experiments.

**Figure 3:**
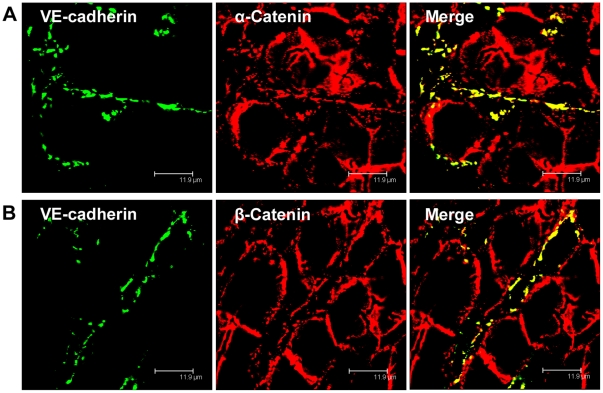
α-Catenin and β-Catenin co-localize with VE-cadherin in human LSECs. Immunofluorescent co-staining of human liver cryosections with anti-VE-cadherin (A, B, green), anti-α-Catenin (A, red), and anti-β-catenin (B, red) antibodies. Images were acquired using laser scanning confocal microscopy. Bars 11.9 µm.

**Figure 4:**
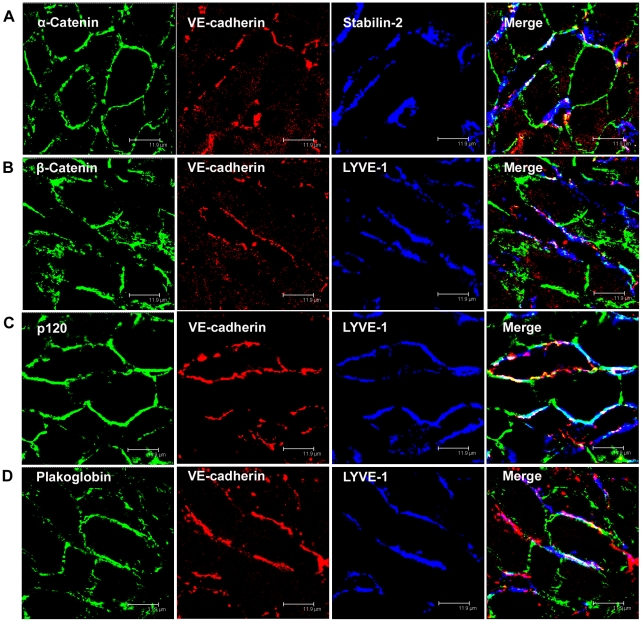
α-catenin, β-catenin, p120-catenin, and plakoglobin co-localize with VE-cadherin in rat liver sinusoidal endothelial cells. (A-D) Immunofluorescent co-staining of rat liver cryosections with anti-α-Catenin (A, green), anti-β-Catenin (B, green), anti-p120-Catenin (C, green), anti-Plakoglobin (D, green), anti-VE -cadherin (A-D, red), anti-Stabilin-2 (A, blue), and anti-LYVE-1 (B-D, blue) antibodies. Images were acquired using laser scanning confocal microscopy. Bars 11.9 µm.

**Figure 5:**
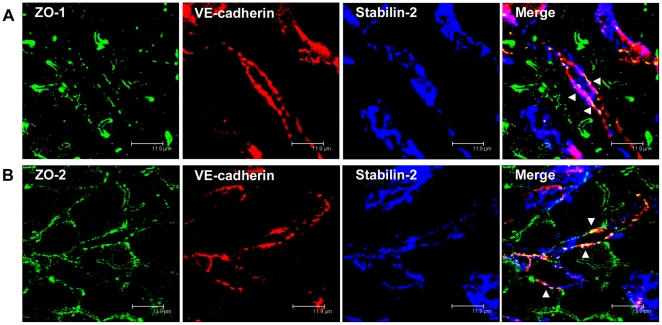
ZO-1 and ZO-2 localize to VE-cadherin-containing cell-cell junctions in rat liver sinusoidal endothelial cells. (A, B) Immunofluorescent co-staining of rat liver cryosections with anti-ZO-1 (A, green), anti-ZO-2 (B, green), anti-VE-cadherin (A, B, red), and anti-Stabilin-2 (A, B, blue) antibodies. Images were acquired using laser scanning confocal microscopy. Bars 11.9 µm.

**Figure 6:**
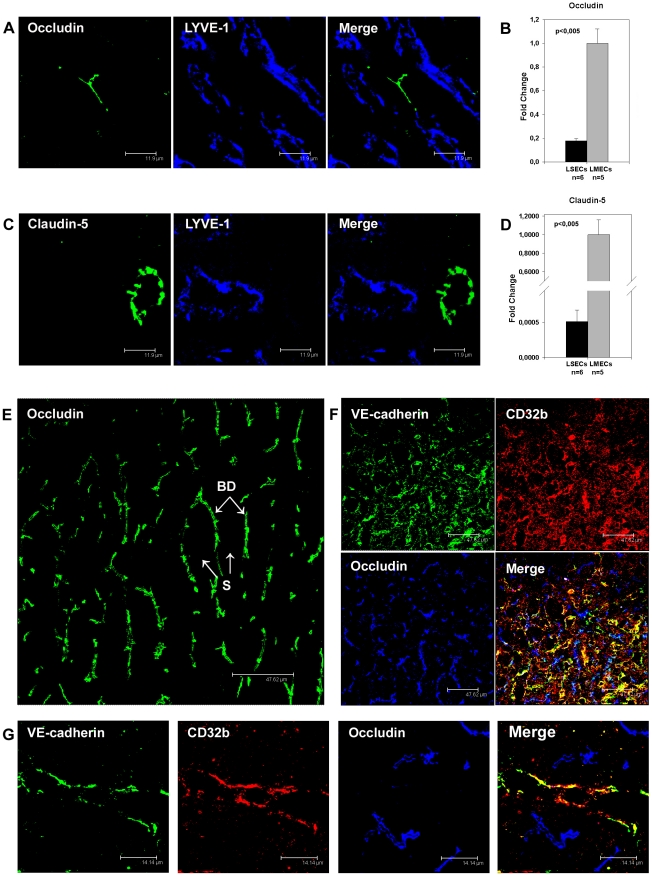
Immunoflurescent and qRT-PCR analysis of occludin and claudin-5 expression in liver sinusoidal endothelial cells. (A, C) Immunofluorescent co-staining of rat liver cryosections with anti-Occludin (A, green), anti-Claudin-5 (C, green), and anti-LYVE-1 (A, C, blue) antibodies. (E) Immunofluorescent staining of a liver sample obtained from the patient 2 with anti-Occludin (green) antibody; BD – bile ducts, S – liver sinusoids. (F, G) Immunofluorescent co-staining of liver samples obtained from the patients 6 (F) and 4 (G) with anti-VE-cadherin (F, G, green), anti-CD32b (F, G, red), and anti-Occludin (F, G, blue) antibodies. Images were acquired using laser scanning confocal microscopy. Bars 11.9 µm (A, C), 47.62 µm (E, F), 14.14 µm (G). (B, D) Quantitative reverse transcriptase-PCR with mRNA isolated from rat LSECs and rat LMECs (n indicates the number of samples analyzed, error bars represent SEM). Primers specific for Occludin (B), Claudin-5 (D), and β-Actin as normalizer were used.

**Figure 7:**
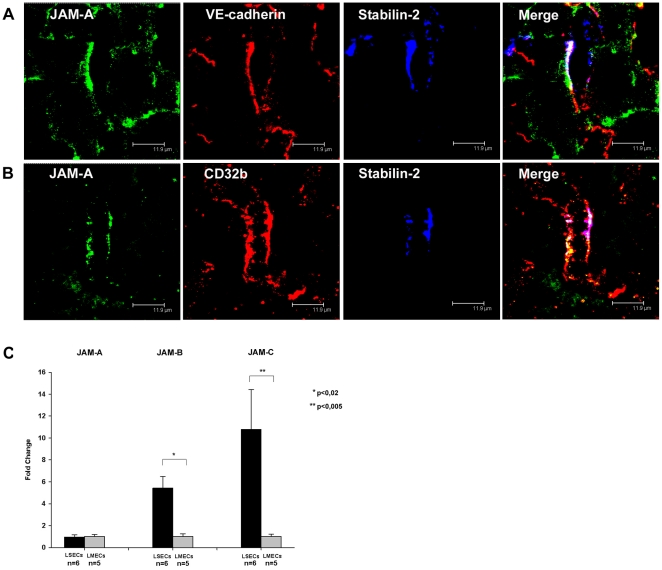
Expression of JAM-family members in liver sinusoidal endothelial cells. (A) Immunofluorescent co-staining of rat liver cryosections with anti-JAM-A (green), anti-VE-cadherin (red), and anti-Stabilin-2 (blue) antibodies. (B) Immunofluorescent co-staining of human liver cryosections with anti-JAM-A (green), anti-CD32b (red), and anti-Stabilin-2 (blue) antibodies. Images were acquired using laser scanning confocal microscopy. Bars 11.9 µm. (C) Quantitative reverse transcriptase-PCR with mRNA isolated from rat LSECs and rat LMECs (n indicates the number of samples analyzed, error bars represent SEM). Primers specific for JAM-A, JAM-B, JAM-C, and β-Actin as normalizer were used.

### Primer Design, cDNA Synthesis, and PCR

Primers listed in Table 3 were designed using Primer-Blast (http://www.ncbi.nlm.nih.gov/tools/primer-blast/). RNA extraction was carried out using the RNeasy Kit (Qiagen, Hilden, Germany). After DNase I (Fermentas, St. Leon-Rot, Germany) pre-treatment, complementary DNA (cDNA) was synthesized with RevertAid H Minus M-MuLV Reverse Transcriptase (Fermentas, St. Leon-Rot, Germany). PCR with DFS-Taq DNA Polymerase (BIORON, Ludwigshafen, Germany) was performed according to the following program: 95°C for 2 min, followed by 27 cycles of 95°C for 15 sec, 60°C for 30 sec, 72°C for 60 sec, followed by final elongation at 72°C for 7 min.

Quantitative PCR (qPCR) were performed with M×3005P QPCR System (Agilent Technologies, Santa Clara, CA) in 10 µl reactions containing 0,8 µM primers, 0,6 µl template DNA, and 5,8 µl SYBR Green PCR Master Mix (Applied Biosystems, Darmstadt, Germany). Cycling conditions were as follows: initial denaturation at 95°C for 10 min, followed by 50 cycles of 95°C for 30 sec; 60°C for 1 min. Data were obtained in triplicates for each sample. Relative gene expression levels were analyzed using SigmaPlot 11 Software (Systat Software GmbH, Erkrath, Germany). To test for statistically significant differences in relative gene expression between LSECs and LMECs, Mann-Whitney Rank Sum Test was applied.

## Results

### VE-cadherin is the Major Cadherin Expressed in Rat and Human LSECs

VE-cadherin expression in human and rat LSECs *in situ* was assessed by immunofluorescent analysis of liver cryosections. In both species, LSECs consistently showed VE-cadherin-positive immunostaining outlining the wall of the hepatic sinuses. Specific expression of VE-cadherin in LSECs was confirmed by double-staining and co-localisation of VE-cadherin using different mono- as well as polyclonal antibodies in conjunction with established LSECs markers [18], [39] such as CD32b and Lyve-1 (Figure 1A, 1B), as well as stabilin-1 and stabilin-2 (data not shown). VE-cadherin was uniformly expressed in the liver sinusoids across all hepatic zones (Figure S1). In addition, isolated stabilin-2 positive rat LSECs kept in culture for 24h displayed prominent VE-cadherin immunoreactions at the intercellular borders as revealed by immunofluorescent analysis (Figure 1C). To further confirm expression of VE-cadherin in LSECs, cDNA of freshly isolated highly pure rat LSECs was analyzed by reverse transcriptase PCR (RT-PCR) with two independent sets of primers specific for VE-cadherin. Both sets of primers showed a similar level of expression of VE-cadherin mRNA in LSECs as compared to lung microvascular endothelial cells (LMECs). In contrast, the rat hepatoma cell line McA-RH7777 used as a negative control did not contain significant amounts of VE-cadherin (Figure 1D). Thus, VE-cadherin expression was clearly demonstrated in rat and human LSECs on the protein and mRNA level.

Rat and human liver sections were also immunostained with antibodies to N-cadherin and E-cadherin to test whether LSECs express additional classical cadherins. E-Cadherin was found to be strongly enriched on the bile canalicular and the lateral cell membrane of hepatocytes. Only minor E-cadherin-positive immunostaining was seen along the space of Disse and there was minimal, if any, overlap with Lyve-1- or VE-cadherin-positive LSECs (Figure 2A, 2C). N-Cadherin was detected at the intercellular contact sites between hepatocytes as well as along the wall of the sinuses (Figure 2B). As the space of Disse is quite narrow in the rat, N-cadherin-positive immunoreactions along the sinuses could not be unequivocally assigned to hepatocytes, hepatic stellate cells or LSECs on tissue sections of rat liver. In the human liver where the space of Disse often appeared wider, there was only incidental co-localisation of N-cadherin with VE-cadherin (Figure 2D). In addition, RT-PCR analysis of freshly isolated LSECs demonstrated strong mRNA expression of VE-Cadherin and only minor mRNA expression of N- and E-Cadherin (Figure 2E). Furthermore N-Cadherin expression was not detectable by immunofluorescent analysis in isolated rat LSECs kept in culture for 2 h, 6 h and 24 h (data not shown). Thus, VE-Cadherin is the major AJ-Cadherin in rat and human LSECs.

### VE-cadherin co-localizes with Catenins and Zonula Occludens Proteins in LSECs

When immunofluorescent double labelling analyses were performed using VE-cadherin antibodies and antibodies against plaque proteins of AJ known to interact with VE-cadherin, VE-cadherin was observed to co-localize with α-catenin and β-catenin in the human liver sinusoids (Figure 3 A and B). Co-localization of VE-cadherin with α- and β-catenin as well as with p120-catenin and plakoglobin was also clearly demonstrated in stabilin-2 and Lyve-1 positive rat LSECs (Figure 4 A-D, Figure S2). Co-occurrence of VE-cadherin with several AJ plaque components in rat and human LSECs suggests the presence of AJ-like protein complexes in LSECs.

Furthermore, triple immunofluorescent analyses were performed with antibodies against the cytoplasmic plaque proteins ZO-1 and ZO-2 which can occur both at AJ and at TJ, in combination with antibodies against VE-cadherin and the LSECs marker stabilin-2. Both, ZO-1 and ZO-2 co-localized with VE-cadherin in rat LSECs *in situ* (Figure 5 A-B).

### Diminished Expression of Tight Junction Transmembrane Proteins Claudin-5 and Occludin and Enhanced Expression of JAM-C in LSECs

To address the question whether typical endothelial TJ molecules are present in LSECs, expression of claudin-5 and occludin was examined in rat LSECs both on protein and mRNA levels. By immunofluorescent analysis of rat liver cryosections, occludin was absent from LYVE-1 positive LSECs, but strongly expressed at the biliary pole of hepatocytes (Figure 6A). Similarly, claudin-5 was absent from VE-cadherin positive rat liver sinusoids, but clearly detectable in endothelial cells of non-sinusoidal hepatic blood vessels (Figure 6C). To confirm these results, quantitative RT-PCR (qRT-PCR) for occludin and claudin-5 mRNA was performed with isolated rat LSECs. Expression of both occludin and claudin-5 was lower in rat LSECs on mRNA level by 5.7 and 1955 fold respectively as compared to LMECs (Figure 6B and D). In human liver samples obtained from three patients without liver fibrosis (Figure 6E) and three patients with liver fibrosis (Figure 6F and G), the bile canaliculi of hepatocytes were strongly positive for occludin while the liver sinusoids were not stained with anti-occludin antibodies. In contrast to rat, human liver sinusoids displayed heterogenous claudin-5 expression varying among different patients as well as among different sections analyzed from the same patient (Figure S3). In contrast to Claudin-5, however, Occludin was consistently absent and VE-Cadherin was evenly expressed in LSECs in all analyzed human tissue samples with differing degrees of hepatic injury. The heterogeneous Claudin-5 expression observed here is in line with a detailed analysis of a larger cohort of patients previously published [26].

JAM-A, -B, and -C are further transmembrane components of TJ reported to be expressed in endothelium. In rat and human liver samples, JAM-A co-localized with stabilin-2 (Figure 7A and B). Furthermore, in rat liver samples, JAM-A was found to co-localize with VE-cadherin (Figure 7A). Upon qRT-PCR for JAM-A, JAM-B and JAM-C, equally strong expression of JAM-A was found in rat LSECs and LMECs (Figure 7C). Interestingly, JAM-B and -C were overexpressed in rat LSECs on mRNA level by 5.4 and 10.8 fold respectively as compared to LMECs (Figure 7C). Thus, LSECs are characterized by lack of significant occludin expression, by a variable Claudin-5 expression and overexpression of JAM-B and JAM-C.

## Discussion

In the present study, we performed a comprehensive analysis of the molecular composition of the intercellular junctional complexes of liver sinusoidal endothelium *in vivo* and *in vitro* in two different species, i.e. human and rat. Our results unequivocally demonstrate that LSECs assemble the molecular complexes typical of endothelial AJ clearly disproving the notion that LSECs lack interendothelial junctions [30]. Similar to vascular endothelial cells in general, LSECs express the main transmembrane component of endothelial AJ, i.e. VE-cadherin, on mRNA and protein level. In freshly isolated rat LSECs, VE-cadherin is found at the intercellular borders in similar localization patterns as in other ECs *in vitro*. Co-localization of VE-cadherin with α-, β-, and p120-catenins, as well as with plakoglobin indicates that LSECs form functional AJ complexes supporting the structural integrity of the sinusoidal vessel wall.

In contrast to most other vascular endothelial cells, the core endothelial TJ proteins claudin-5 and occludin were absent from the rat LSECs as assessed *in vivo* and *in vitro* and on the protein and mRNA level. Similarly, human liver sinusoids lacked occludin expression. However, in human liver samples, we observed heterogenous claudin-5 immunostaining in LSECs while non-sinusoidal liver vessels exhibited strong claudin-5 immunoreactivity. Previously, Sakaguchi and colleagues also demonstrated heterogeneous Claudin-5 expression in tumoral endothelium of hepatocellular carcinoma and surrounding LSECs depending on fibrotic grade and tumor differentiation [26]. Thus the differences in claudin-5 expression between LSECs of healthy young rats and human livers may be due to the fact that the human liver samples available for investigation were derived from older patients that had succumbed to liver disease. This may have caused capillarization of liver sinusoids characterized by quantitative and qualitative changes of cell-cell and cell-matrix adhesion molecules in LSECs [9], [15], [23], [40]. Thus, plasticity of the LSECs phenotype upon induction of capillarization may be one possible explanation for the claudin-5 immunoreaction found in human but not in rat liver sinusoids. Of course, the expression of claudin-5 in human LSECs *in situ* may also be due to true species-specific differences. The notion that claudin-5 and occludin are dispensable for LSECs function is supported by findings in occludin and claudin-5 knock-out mice which display normal vascular development, structure and function in the liver, but show increased vascular permeability in the cerebral vasculature [41], [42].

While lacking some of the typical transmembrane constituents of endothelial TJ, we show here that LSECs express the zonula occludens proteins ZO-1 and ZO-2. ZO-1 was first identified as an intracellular partner of transmembrane components of TJ such as claudins and occludin, but it has also been found in AJ at the early stage of establishing endothelial cell-cell contacts when TJ had not yet been formed [31]. In addition, in cells lacking TJ such as fibroblasts and cardiomyocytes, ZO-1 localized constitutively to cadherin-containing intercellular junctions, i.e. *bona fide* adherens junctions [35]. Furthermore, it has been shown that ZO-1 may localize to both TJ and AJ concomitantly and is indispensable for the formation of both types of junctions [34].

In addition, we show here that LSECs express all members of the family of junctional adhesion molecules, i.e. JAM-A, -B, and –C which are known to participate in the formation and dynamics of different types of intercellular contacts [28], [43]. In this study, we do not only show expression of JAM-C in LSECs, but we demonstrate by using qRT-PCR that JAM-B and JAM-C expression is much stronger in isolated rat LSECs as compared to LMECs. This finding may be of potential importance as Orlova and colleagues have shown that shifting the balance from JAM-A to JAM-C promotes destabilization of VE-cadherin-mediated interendothelial adhesion and increases vascular permeability [36]. Owing to heterophilic interactions between JAM-A, JAM-B, and JAM-C and the leukocyte integrins LFA-1, VLA-4, and Mac-1, respectively, JAMs may also contribute to transmigration of leukocytes and tumor cells through the liver-specific transendothelial barrier established by LSECs. Interestingly, murine heart endothelial cells have been found to facilitate selective recruitment of Th1 lymphocytes upon stimulation with TNF-α in contrast to murine lung endothelial cells [44]. This effect is only partially due to interactions between VCAM-1 and VLA-4 evoking the hypothesis that enhanced JAM-C expression in murine heart endothelial cells could play a decisive role. Thus, JAMs expressed by LSECs may be involved in the recruitment of specific subpopulations of lymphocytes as well as of tumor cells to the liver parenchyma.

Taken together, our data prove the existence of organ-specific intercellular junctions between endothelial cells of the liver sinusoids. These junctions contain the full repertoire of proteins typical of AJ, i.e. VE-cadherin, catenins and plakoglobin, and the facultative AJ proteins ZO-1 and ZO-2. Interestingly, these AJ proteins co-occur with the JAM-family members, but not with the core TJ proteins claudin-5 and occludin. The composition of the intercellular junctions between LSECs molecularly resembles that of the intercellular junctions between high endothelial venules (HEV) representing a site for lymphocyte homing from the blood to the lymphoid tissues [33]. Nevertheless, the junctions of HEV EC differ from those of LSECs in their ultrastructure as revealed by electron microscopy [45], [46] indicating so far unrecognized differences also in the molecular architecture of the junctional complexes of these two types of endothelial cells. Therefore, it is well conceivable that the junctions described here represent a unique kind of mixed-type junction. In summary, the present study contributes to the currently accumulating knowledge about cell-type specific intercellular junctions between endothelial cells of different microvascular beds. The comprehensive molecular characterization of the specialized intercellular junctions between LSECs provides a framework for further functional investigations of the transendothelial barrier of liver sinusoids in numerous pathological conditions ranging from hepatic inflammation to formation of liver metastasis.

## Supporting Information

Figure S1 **The expression of VE-cadherin in rat liver sinusoidal endothelial cells is not restricted to a particular hepatic zone.** Immunofluorescent co-staining of rat liver cryosections with anti-VE-cadherin (green), anti-CD32b (red), and anti-Stabilin-2 (blue) antibodies. Images were acquired using laser scanning confocal microscopy. Bars 150 µm.
(TIF)

Figure S2 **Intensity correlation analysis of co-localization of VE-cadherin with α-catenin, β-catenin, p120-catenin, and plakoglobin in rat liver sinusoids.** Merged confocal images from the Figure 4 are shown along with positive PDM values (Products of the Differences from the Mean) calculated for each indicated channel pair.
(TIF)

Figure S3 **Heterogenous expression of Claudin-5 in human liver sinusoids.** (A-C) Liver samples obtained from the patients 4 (A, B) and 6 (C) were co-stained with anti-VE-cadherin (green), anti-CD32b (red), and anti-Claudin-5 (blue) antibodies. Images were acquired using laser scanning confocal microscopy. Bars 56.55 µm (A, B), 47.62 µm (C).
(TIF)
