# Supplementary figures and images for: Unique Cell Type-Specific Junctional Complexes in Vascular Endothelium of Human and Rat Liver Sinusoids
Source: PLoS One. 2012 Apr 3;7(4):e34206. doi: 10.1371/journal.pone.0034206 (PMC3317944; doi:10.1371/journal.pone.0034206)

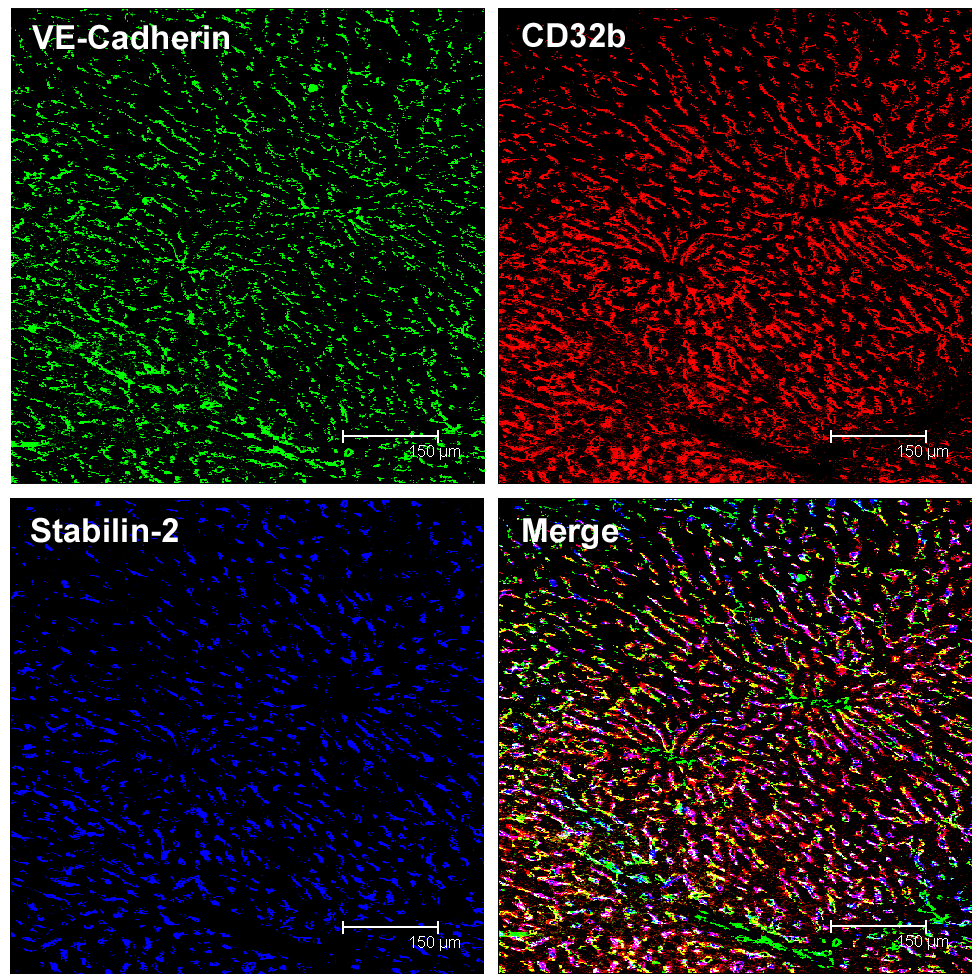

Supplement: Figure S1 — The expression of VE-cadherin in rat liver sinusoidal endothelial cells is not restricted to a particular hepatic zone. Immunofluorescent co-staining of rat liver cryosections with anti-VE-cadherin (green), anti-CD32b (red), and anti-Stabilin-2 (blue) antibodies. Images were acquired using laser scanning confocal microscopy. Bars 150 µm. (TIF) [file pone.0034206.s001.tif]

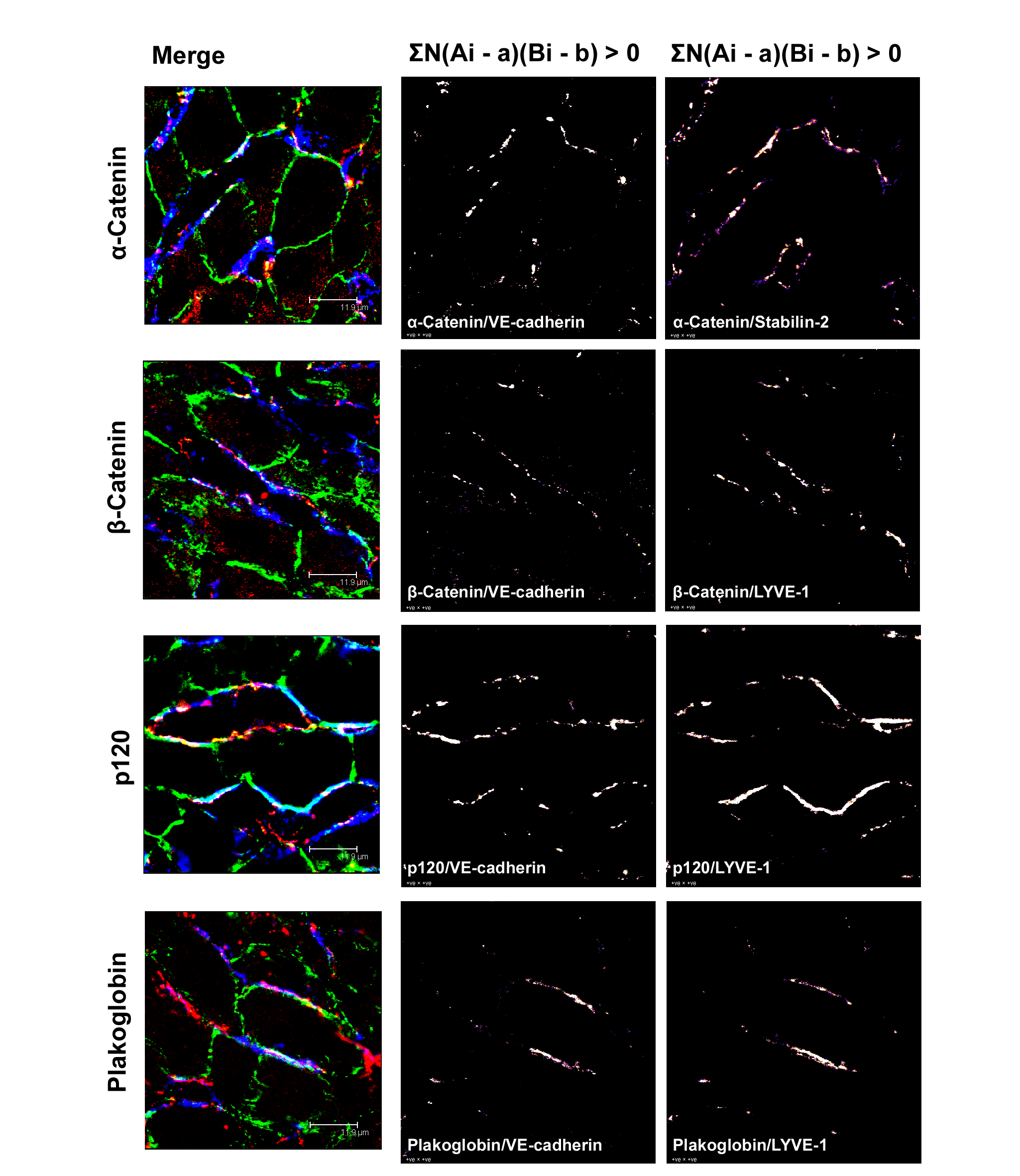

Supplement: Figure S2 — Intensity correlation analysis of co-localization of VE-cadherin with α-catenin, β-catenin, p120-catenin, and plakoglobin in rat liver sinusoids. Merged confocal images from the Figure 4 are shown along with positive PDM values (Products of the Differences from the Mean) calculated for each indicated channel pair. (TIF) [file pone.0034206.s002.tif]

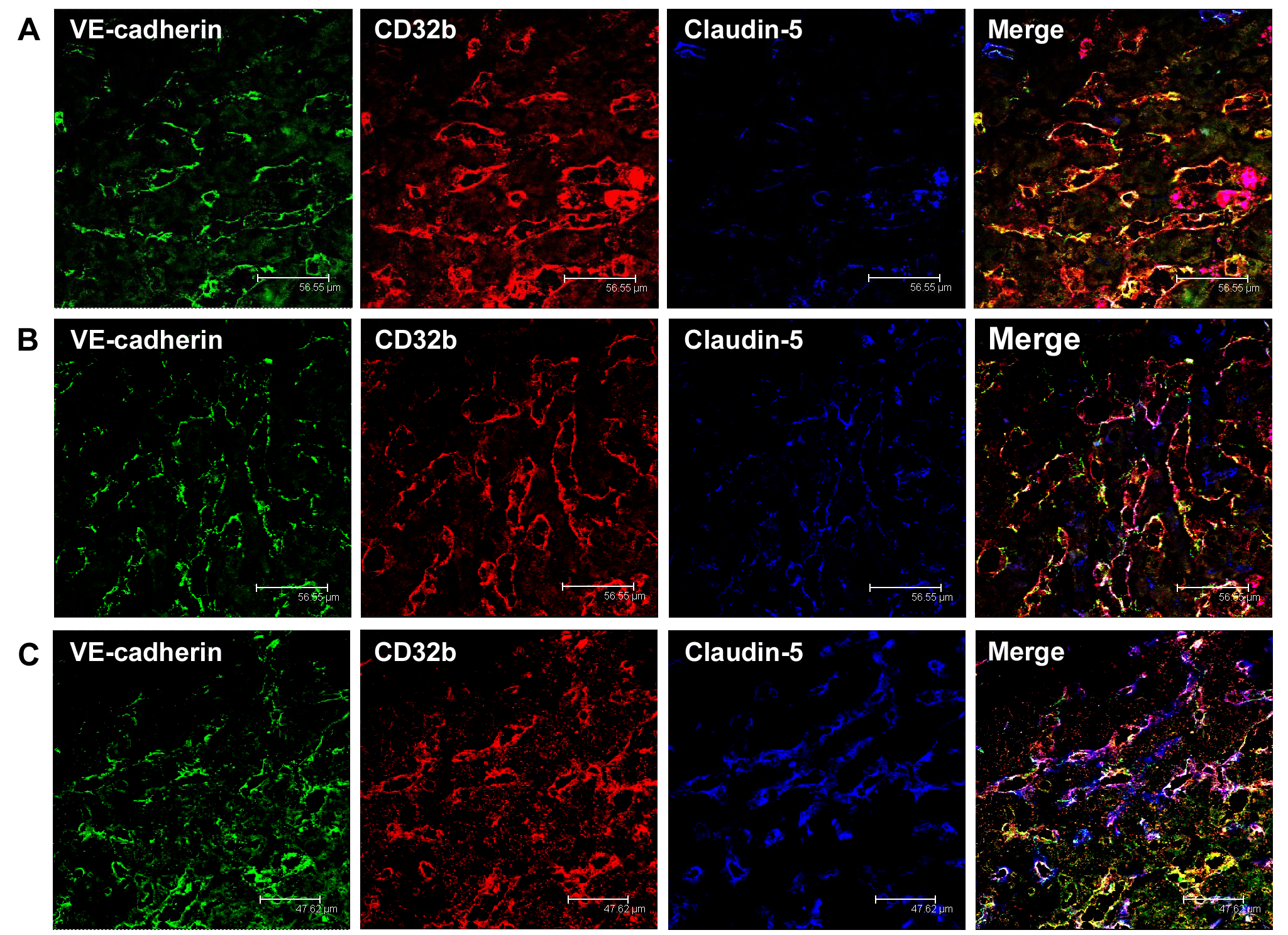

Supplement: Figure S3 — Heterogenous expression of Claudin-5 in human liver sinusoids. (A-C) Liver samples obtained from the patients 4 (A, B) and 6 (C) were co-stained with anti-VE-cadherin (green), anti-CD32b (red), and anti-Claudin-5 (blue) antibodies. Images were acquired using laser scanning confocal microscopy. Bars 56.55 µm (A, B), 47.62 µm (C). (TIF) [file pone.0034206.s003.tif]
